# Dizzy People Perform No Worse at a Motor Imagery Task Requiring Whole Body Mental Rotation; A Case-Control Comparison

**DOI:** 10.3389/fnhum.2013.00258

**Published:** 2013-06-06

**Authors:** Sarah B. Wallwork, David S. Butler, G. Lorimer Moseley

**Affiliations:** ^1^Sansom Institute for Health Research, University of South Australia, Adelaide, SA, Australia; ^2^Neuro Orthopaedic Institute, Adelaide, SA, Australia; ^3^Neuroscience Research Australia, Sydney, NSW, Australia

**Keywords:** motor imagery, dizziness, left/right judgments, vestibular, implicit movements

## Abstract

We wanted to find out whether people who suffer from dizziness take longer than people who do not, to perform a motor imagery task that involves implicit whole body rotation. Our prediction was that people in the “dizzy” group would take longer at a left/right neck rotation judgment task but not a left/right hand judgment task, because actually performing the former, but not the latter, would exacerbate their dizziness. Secondly, we predicted that when dizzy participants responded to neck rotation images, responses would be greatest when images were in the upside down orientation; an orientation with greatest dizzy-provoking potential. To test this idea, we used a case-control comparison design. One hundred and eighteen participants who suffered from dizziness and 118 age, gender, arm pain, and neck pain-matched controls took part in the study. Participants undertook two motor imagery tasks; a left/right neck rotation judgment task and a left/right hand judgment task. The tasks were completed using the Recognise program; an online reaction time task program. Images of neck rotation were shown in four different orientations; 0°, 90°, 180°, and 270°. Participants were asked to respond to each “neck” image identifying it as either “right neck rotation” or a “left neck rotation,” or for hands, a right or a left hand. Results showed that participants in the “dizzy” group were slower than controls at both tasks (*p* = 0.015), but this was not related to task (*p* = 0.498). Similarly, “dizzy” participants were not proportionally worse at images of different orientations (*p* = 0.878). Our findings suggest impaired performance in dizzy people, an impairment that may be confined to motor imagery or may extend more generally.

## Introduction

Dizziness is common in people with neck pain – about 15% of people with Whiplash Associated Disorder (WAD) complain of dizziness within the first week after injury (Sterner and Gerdle, 2004). In addition to pain, several pathological processes can contribute to the perception of dizziness, the most common being vestibular disorders, followed by psychiatric disorders and presyncope (Kroenke et al., 1992). Dizziness often has multifactorial etiologies (Hoffman et al., 1999). One’s perception of dizziness can be triggered by alterations of incoming sensory input, integration of these sensory inputs, or changes to the effector organs themselves (Luxon, 2004). Therefore, moving one’s body into different orientations can be aggravating for the dizzy patient, as it triggers an influx of sensory information from the sensory organs.

It has been well established that a motor imagery task of making left/right judgments of body parts activates similar cortical areas to those activated for the actual or imagined movement (Parsons, 2001). Left/right body part judgment tasks require one to look at an image of a body part and identify it as either belonging to the left or right side of the body (i.e., hands or feet), or rotated or angled toward the left or right side of the body (i.e., neck or trunk rotation). It is thought that the process of choosing a side requires one to access cortical maps associated with the relevant body part and mentally maneuver that body part into the orientation seen in the image, thus revealing whether or not the initial judgment was correct. If the initial side of choice is wrong, the same process will be rerun but for the other side (Parsons, 2001).

Early investigations into the neurological processes involved in making left/right judgments of hands, found that responses were delayed if a high degree of mental rotation was required to match the orientation in the stimulus image (Parsons, 2001). That is, the time taken to perform a mental movement of rotating the hand from its current orientation during the task into the position of the stimulus hand is governed by the normal biomechanical constraints of moving one’s actual hand into the position of the stimulus. In left/right judgments, this is usually undertaken as an implicit movement. That is, we do it without consciously thinking about it. Conversely, an explicit movement is an imagined movement that is consciously thought about. If then, a large and complex movement would be required to match the stimulus hand, a longer response time would be expected, whether or not the participant knew that they were mentally making such a movement. Similarly, in more recent studies looking at left/right neck rotation judgments (Wallwork et al., 2013) and left/right trunk rotation judgments (Bowering et al., 2012), response times were longer when the image was orientated upside down or tilted on the side, and shorter when images were not rotated at all. This finding, and the idiosyncratic pattern of reaction times (Bowering et al., 2012; Wallwork et al., 2013) suggests that left/right judgments of the neck and trunk also require implicit mental rotation of the whole body into a position that matches the image, before being able to make a left/right judgment response. For example, if an image shows a person with their neck rotated to their left, and the image itself is rotated 180°, one might expect that the participant would need to mentally rotate their entire body into the upside down position prior to identifying it as a left-sided neck rotation.

This established relationship between reaction time and “awkwardness” (Moseley, 2004) of the movement is dominated in people with arm pain, by a stronger relationship between reaction time and predicted pain on that movement (Moseley, 2004). That clearly shows that evaluative processes interfere with implicit motor imagery just as they do with executed movements. On the basis of these findings, one would predict that the dizzy patient would perform worse on motor imagery tasks requiring whole body mental rotation because moving the head often exacerbates dizziness in these people. Specifically, we would expect to see a delayed response because we would predict that people who suffer from dizziness would avoid those movements in much the same way that chronic pain patients show a delayed response to motor imagery of movements that would be painful (Moseley, 2004; Meulders et al., 2011).

We hypothesized that participants who reported dizziness would take longer to complete a left/right neck rotation judgment task than they would a left/right hand judgment task, when compared to healthy age, gender, and pain-matched controls. Our secondary hypothesis was that the delay in response time would be greatest for images of upside down orientation; that is, dizzy participants would take longer to respond to images that required implicitly turning oneself upside down.

## Materials and Methods

### Design

A case-control comparison design was used.

### Participants

Data were obtained from a large cross-sectional study previously undertaken investigating left/right neck rotation judgments and left/right hand judgments (Wallwork et al., 2013). These data included 1737 participants from 40 countries, aged between 10 and 90 years, both males and females. Participants were recruited through social media strategies and by word of mouth, and were asked to complete the study online via a web connected computer. All participants volunteered their time and were able to withdraw at any point. Ethical approval was granted by the institutional ethics committee.

### Questionnaire

Prior to undertaking the left/right judgment tasks, participants were asked to complete a questionnaire regarding their demographic details, physical activity, presence of pain, and general health. Included in this questionnaire was a question relating to whether or not the participant suffers from dizziness; we asked, “Do you suffer from dizziness?” Participants were required to tick either a “yes” or a “no” box.

### The tasks

In total there were three motor imagery tasks. Each task involved making responses to a different batch of 40 photographs. The first task required participants to respond to plain photographs of someone turning their head to one side, and respond to each as being either a left neck rotation or a right neck rotation. This task was repeated three times; the first run within this first task was considered a practice, the second run was used for analysis in the current study, and the third run was considered to be influenced by fatigue, and therefore not included in the analysis. The second task also required left/right neck rotation judgments, however the photographs were taken of people in contextually variable environments and was also not analyzed in the present study. The third task was a left/right hand judgment task. We did not include a practice run for this third task because previous data suggests that performance would be similar. This was confirmed when we compared the current results to previously published results (Wallwork et al., 2013). Responses were made using the “a” key for a left-sided response and “d” for a right-sided response. When a response was made, the next image would immediately appear. If no response was made, or if the participant did not respond within 5 s, the next image would appear and the time was recorded as 5 s for that image and a blank response would be displayed in place of a “left” or a “right” response. Response times were taken from all responses, not just correct responses, meaning that in the unlikely occasion that participants responded in exactly 5 s, we were not able to distinguish between them and people who were not able to respond within the allocated time frame.

### The photographs

Each task displayed a separate batch of 40 images (20 female). The first task displayed portrait images of a person wearing a black t-shirt with their head rotated to either their left or their right side, relative to their shoulders. There were equal numbers of left and right neck rotations, at 0°, 90°, 180°, and 270° of whole image rotation. The photographs were taken from either a front, back, or side view, and all participants viewed and responded to the same images. In the second task, photographs were taken of a range of people in different environments. This batch of images was not used for analysis in the current study. The third and final task displayed photographs of hands in varying postures with either a black, white, or green background. Only the hand, wrist, and distal arm were in view. We chose not to orientate the hand images as we did the neck images, on the basis that the hands have greater degrees of freedom than the neck, and rotating the image would not have the clear demarcation of rotation that we see for images of the neck.

### Data cleaning

Prior to analysis, a total of 1792 data sets were “cleaned.” This involved complete data sets being removed if the questionnaire had not been filled out or if all tasks were not finished; 55 data sets were removed for these two reasons, leaving 1737 complete data sets. Single responses were also removed if they were <500 ms, which we took to be too fast for a true judgment response (Kunde, 2001), or if they timed out for eight or more (≥20%) images in a row, which we took to be a distraction from the task or computer malfunction.

**Figure 1:**
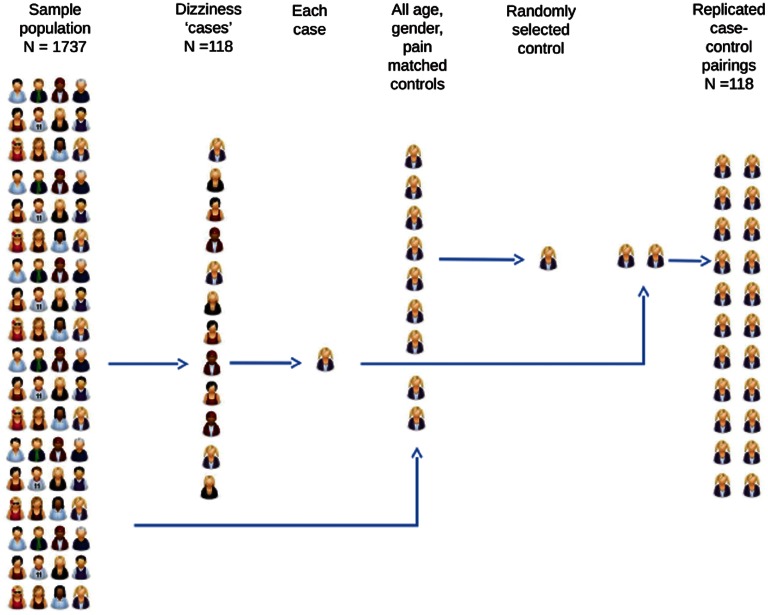
**Flow diagram detailing the case-control comparison design**. Participants in the sample population who reported dizziness were identified and allocated to the “dizzy” group. Age, gender, and pain-matched controls were randomly selected from the remaining sample population.

Participants who had reported in the questionnaire that they experience dizziness, were selected and allocated to the “dizzy” group. A control participant was randomly selected from the pool of participants who matched each dizzy participant for age, gender, neck pain, and arm pain. We did not ask participants about any other areas of pain, and therefore did not match for this. The participants identified in this process were allocated to the control group (see Figure 1 for flow chart). Pain is known to affect performance in left/right judgment tasks (Moseley, 2004; Bray and Moseley, 2011; Bowering et al., 2012; Leake et al., unpublished data).

### Data analysis

Data were analyzed using SPSS 19.0. Descriptive statistics were first collated to get an idea of the sample population. Two repeated measures ANOVAs (response time and accuracy) were run to see whether people who reported suffering from dizziness performed differently to controls at the two left/right judgment tasks. For each ANOVA there were two factors; within-subjects (hand judgments or neck judgments) and between subjects (dizziness or no dizziness). To see whether there was an effect of image rotation during the left/right neck rotation task, another repeated measures ANOVA was conducted, again with two factors; within-subjects (image rotation – four levels) and between subjects (dizziness or no dizziness). Significance was set at α = 0.05.

## Results

### Participants

One hundred eighteen participants (104 females) reported symptoms of dizziness. Thirty four (28%) reported neck pain and 23 (19.5%) reported arm pain. Mean age was 42 (SD 13) years. This group was matched for age, gender, neck pain, and arm pain to yield a cull cohort of 236 participants.

### Dizziness versus no dizziness

Response times and accuracies for the two groups are in Table 1.

**Table 1:** **Response time and accuracy for left/right neck rotation judgments and left/right hand judgments in people with and without dizziness symptoms**.

	Neck judgments		Hand judgments	
	
	
	Response time (ms ± SD)	Accuracy (% ± SD)	Response time (ms ± SD)	Accuracy (% ± SD)
“Dizzy” group	1786.7 ± 516.3	88.0 ± 12.3	2096.9 ± 569.7	86.3 ± 11.0
Control group	1617.9 ± 455.7	88.5 ± 13.2	1974.1 ± 550.5	87.4 ± 10.9

### Response time

There was a main effect of dizziness on response times. Participants in the “dizzy” group were slower at both tasks than controls [*F*(1,234) = 6.032, *p* = 0.015]. Mean difference and 95% confidence interval was 145.81 and 28.8–262.7 ms, respectively. Regardless of group, all participants were faster at left/right neck rotation judgments than they were at left/right hand judgments [*F*(1,234) = 97.084, *p* < 0.001], however there was no Dizzy × Task interaction (*p* = 0.498). That is, dizzy people were slower than the controls, but they were no more delayed in their responses for the neck judgment task than they were for the hand judgment task.

### Effect of image orientation on the left/right neck rotation task

**Figure 2:**
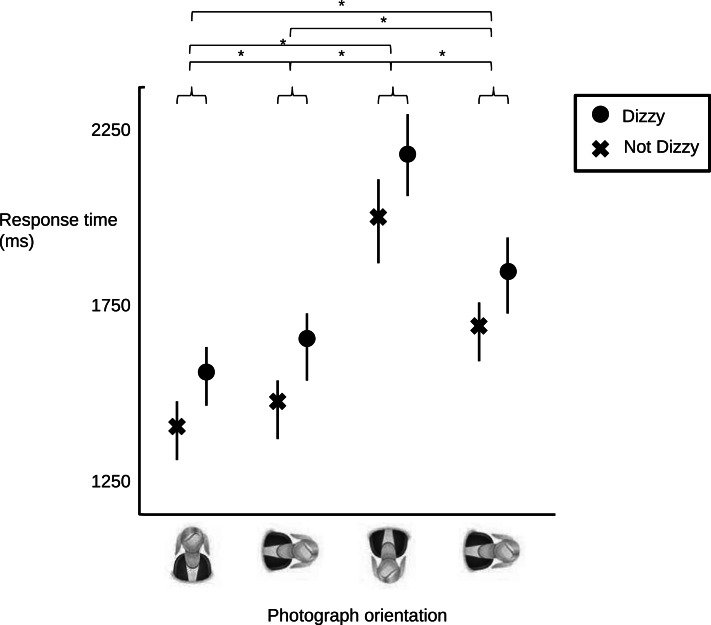
**Mean (circles and crosses) and 95% confidence intervals (lines) for response times for the “dizzy” group (circles) and control group (crosses) at the four image orientations**. There was no Group × task interaction, but there was an effect of orientation. Asterisk (*) denotes significant at *p* < 0.01.

Consistent with the above results, there was an effect of dizziness on response times [*F*(1,234) = 7.115, *p* = 0.008]. Mean differences and confidence intervals for the four image orientations are given in Table 2. There was an effect of image orientation [*F*(3,702) = 203.65, *p* < 0.001] (see Figure 2), but there was no Dizzy × Orientation interaction (*p* = 0.878). That is, dizzy people were no more delayed in their responses for the neck judgment task when the task required full body rotation than when it did not.

**Table 2:** **Mean. differences and 95% confidence intervals for the four image orientations**.

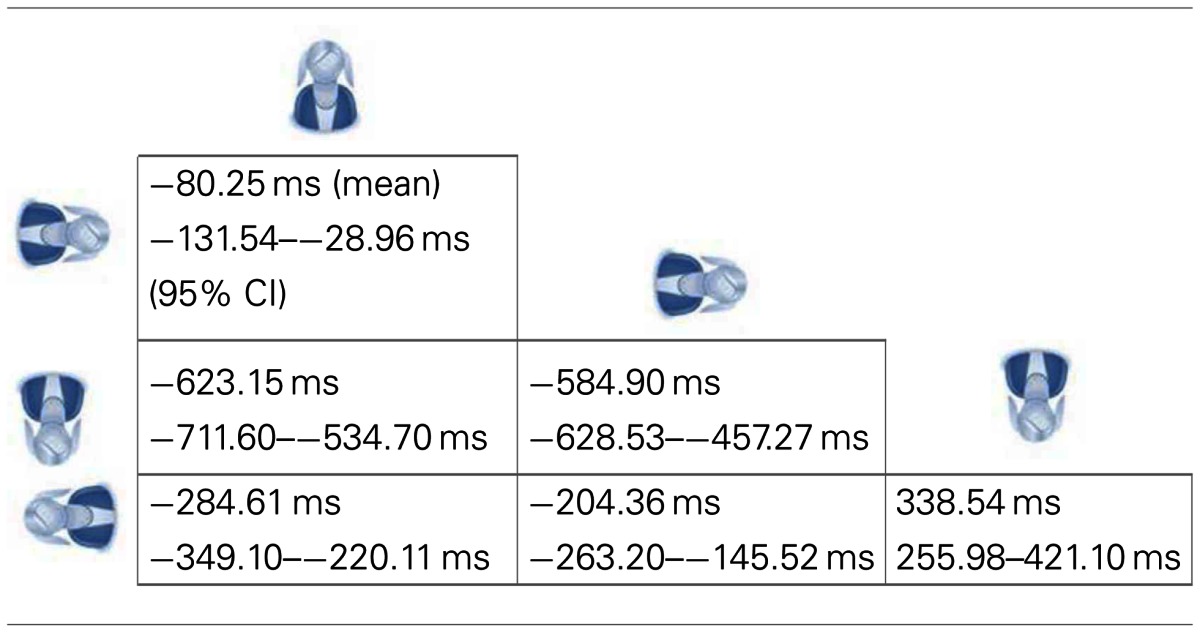

### Accuracy

There was no main effect of dizziness on accuracy scores. Participants in the “dizzy” group were no more or less accurate at either task [*F*(1,234) = 0.364, *p* = 0.547]. Regardless of group, participants were no more or less accurate at one task over another [*F*(1,234) = 3.167, *p* = 0.076], and again there was no Dizzy × Task interaction (*p* = 0.673).

## Discussion

The results of the current study did not support our hypothesis that in an online study, people who report dizziness, response time would be longer for a left/right neck rotation judgment task, but not a left/right hand judgment task, than it is for non-dizzy age, gender, and pain-matched controls. Nor did our results support our secondary hypothesis that the delay in response in dizzy people would be greatest for images that required full body rotation. Instead, we found that participants from the “dizzy” group were slower than controls at both tasks and the extent to which they were worse was not affected by image rotation.

Importantly, the majority of our participants were female (104 out of 118 participants). This stark imbalance was surprising. We have previously found that females take longer to respond to images in a left/right neck rotation judgment task than males (Wallwork et al., 2013), and that we controlled for gender in the current analysis helps us to account for this difference. Although we have no reason to suspect that females and males with and without dizziness would demonstrate differential results, it would seem imprudent to generalize the results to males before the finding is replicated with a greater representation of males.

Our results did show that participants took longer to respond to images with a high degree of rotation which would be expected based on previous studies of the hand (Parsons, 2001; Schwoebel et al., 2001; Moseley, 2004), neck (Wallwork et al., 2013), and trunk (Bowering et al., 2012), as the mental movement to match the stimulus would take longer when one is required to turn themselves upside down. However, we also expected that participants suffering from dizziness would take proportionally more time to respond to images rotated 180°, due to a dizzy-avoidance type behavior. That we did not find this suggests that people who suffer from dizziness either do not implicitly avoid dizziness-provoking movements, or may employ a different strategy by which to perform the task. Perhaps people with dizziness rotate the picture rather than their own body (Dey et al., 2012) to overcome this problem. Unfortunately the current study was not equipped to investigate potential mechanisms involved here.

That participants in the “dizzy” group were slower than controls at both left/right judgment tasks further reinforces that it probably is not the implicit whole body rotation that affected performance. This raises the possibility that people who suffer from dizziness are worse at motor imagery, or indeed, worse at choice reaction tasks. Evidence suggests that people with vestibular dysfunction may have mild cognitive impairment (Smith et al., 2005); being both spatial and non-spatial in nature. The current study utilized only spatial tasks and to include a non-spatial task may have shed light on this possibility.

We cannot discount the potential for an order effect confounding the current findings. Although an important limitation, it seems unlikely that an order effect has contributed to the current findings, because if it did we should have seen a decline in accuracy scores, and response times slower than those obtained previously for identical tasks (Moseley, 2004; Hudson et al., 2006), neither of which we observed.

The role of the vestibular apparatus in spatial representation does appear to be important. It has been recognized that people with vestibular syndromes have poor spatial awareness (Borel et al., 2008). The vestibular system plays a key role in multisensory integration and information processing pathways, and allows one to stabilize their gaze, and orientate their head and body in space; hence the vestibular system is necessary in establishing internal representations of body position and body in space. It makes sense then, that people with vestibular disturbances would perform worse at a task requiring body and near-body spatial attention, such as in the left/right judgment tasks. That vestibular disturbances are the most common cause of dizziness (Kroenke et al., 1992) allows us to presume the same applied to our group, but importantly, we cannot be sure. However, people with vestibular dysfunction can include people with and without symptoms of dizziness, and people with dizziness can include people with and without vestibular disturbances (Kroenke et al., 1992), so at this stage we cannot verify this presumption. Further investigations in people with vestibular causes of dizziness would need to be conducted to test this idea.

A final and unsurprising finding of the current study was that participants in the “dizzy” group were no more or less accurate than those in the control group. Reduced accuracy is more likely to be due to imprecise cortical proprioceptive representation of the relevant body part (Bray and Moseley, 2011) and as such we would not predict a difference between dizzy and non-dizzy people. We do see reduced accuracy of left/right judgments in people with phantom limb pain (Nico et al., 2004), back pain (Bray and Moseley, 2011; Bowering et al., 2012), and neck pain (Leake et al., unpublished data), but in each case there is clear evidence of disruption of cortical proprioceptive representation (see Wand et al., 2011; Moseley et al., 2012 for reviews).

In summary, our results did not support the hypotheses that, in people who report dizziness, response time would be longer for a left/right neck rotation judgment task, but not a left/right hand judgment task, and that the delayed response in dizzy people would be greatest for images that required full body rotation. Participants in the “dizzy” group were not proportionally worse at responding to images thought to require implicit whole body rotation and that we expected would provoke dizziness if they were performed. Importantly, our participants are largely representative of females which needs to be acknowledged as it has the potential to create bias in the current results. Our results do suggest that dizziness might be associated with cognitive impairment, poor spatial processing, or poor motor imagery.

## Conflict of Interest Statement

David S. Butler is Managing Director of the Neuro Orthopaedic Institute who sells Recognize, the program used to record data for this study.
